# Supplementary material for: A Missense Variation in PHACTR2 Associates with Impaired Actin Dynamics, Dilated Cardiomyopathy, and Left Ventricular Non-Compaction in Humans
Source: Int J Mol Sci. 2023 Jan 10;24(2):1388. doi: 10.3390/ijms24021388 (PMC9864900; doi:10.3390/ijms24021388)
Supplement: Supplementary file 1 [file ijms-24-01388-s001.zip › Supplementary.pdf]

Supplementary Table S1: Clinical evaluation of II-3 family members. The annotation of the individuals is according to Fig. 1A. Abbreviations: LVESD: left ventricular end-systolic diameter, LVEDD: left ventricular end-diastolic diameter (adult norm: 35-54mm), EF: ejection fraction. BSA: body surface area, NSR: normal sinus rhythm.

Patient	Sex	Age	LVEDD (mm) z-score	LVESD (mm)	IV-SEPTUM (mm)	Posterior Wall (mm)	EF%	BSA (M2)	ECG
**I-1**	Male	Mid 30s	51	30	10	8	65%	1.77	NSR No LVH
**I-2**	Female	Mid 30s	50	32	9	9	62%	1.92	NSR No LVH
**II-1**	Male	Teens	44 z-score +0.07	29	8	8	67%	1.43	NSR No LVH
**II-2**	Male	Early teens	39 z-score -0.47	26	5.5	5.7	60%	1.05	NSR No LVH
**II-4**	Female	Infant	21 z-score -2.94	13	3.5	3.5	64%	0.47	NSR No LVH
**II-5**	Male	Early teens	34 -0.38	21	5	5	65%	1/02	NSR No LVH
**II-6**	Male	Infant	24 z-score -2.7	11	4	4	65%	0.579	NSR No LVH

Supplementary Table S2: List of the 47 genes reported to have mutations causing familial and non-familial DCM.

Gene	Protein	OMIM	Reference*
ABCC9	SUR2A	601439	[10,30]
ACTC1	Cardiac actin	102540	[9,10,30]
ACTN2	Alpha-actinin-2	102573	[10,30]
ANKRD1	Ankyrin repeat domain-containing protein 1	609599	[10,30]
BAG3	BCL-associated athanogene 3	603883	[9,10,30]
CAV3	Caveolin 3	601253	[10]
CRYAB	Alpha B crystalin	123590	[10,30]
CSRP3	Muscle LIM protein	600824	[9,10,30]
DES	Desmin	125660	[9,10,30]
DSC2	Desmocollin 2	125645	[9,30]
DSG2	Desmoglein 2	125671	[9,30]
DSP	Desmoplakin	125660	[9,30]
EMD	Emerin	300384	[9,10]
EYA4	Eyes-absent 4	603550	[30]
FLNC	Filamin C	102565	[9,30]
ILK	Integrin-linked kinase	602366	[30]
LAMA4	Laminin alpha-4	600133	[30]
LAMP2	Lysosomal associated membrane protein 2	309060	[10]
LDB3	Cypher/ZASP	605906	[10,30]
LMNA	Lamin A/C	150330	[9,10,30]
MYBPC3	Myosin-binding protein C	600958	[10,30]
MYH6	Alpha-myosin heavy chain	160710	[10,30]

MYH7	Beta-myosin heavy chain	160760	[9,10,30]
MYL2	Myosin light chain 2	160781	[10]
MYL3	Myosin light chain 3	160790	[10]
MYPN	Myopalladin	608517	[9,10,30]
NEBL	Nebulette	605491	[10,30]
NEXN	Nexilin	613121	[10,30]
PDLIM3	PDZ LIM domain protein 3	605889	[30]
PKP2	Plakophilin 2	602861	[30]
PLN	Phospholamban	172405	[9,10,30]
PRKAG2	Protein kinase AMP-activated non-catalytic subunit gamma 2	602743	[10]
RBM20	RNA binding protein 20	613171	[9,10,30]
RYR2	Ryanodine receptor 2	180902	[9]
SCN5A	Sodium channel	600163	[9,10,30]
SCO2	Synthesis of cytochrome c oxidase 2	604272	[10]
SGCD	Delta-sarcoglycan	601411	[10,30]
SURF1	SURF1 cytochrome c oxidase assembly factor	185620	[10]
TAZ	Tafazzin	300394	[10]
TCAP	Titin-cap or telethonin	604488	[10,30]
TNNC1	Cardiac troponin C	191040	[9,10,30]
TNNI3	Cardiac troponin I	191044	[9,10,30]
TNNT2	Cardiac troponin T	191045	[9,10,30]
TPM1	Alpha-tropomyosin	191010	[10,30]
TTN	Titin	188840	[9,10,30]
TTR	Transthyretin	176300	[10]
VCL	Metavinculin	193065	[9,10,30]

OMIM: Online Mendelian Inheritance in Man. DCM, dilated cardiomyopathy; the reference numbers relate to the numbers in the main text.

Supplementary Table S3: Negation of the potential heterozygous candidate variants in genes considered clinically relevant for dilated cardiomyopathy (Supplementary Table 2). The family member's annotations are according to figure 1. Positions on chromosomes are according to GRCh37/hg19.

**Negated by segregation analysis of healthy family members presenting variation in heterozygosity**						
**No**	**Gene**	**Position dbSNP**	**Change**	**Effect**	**ACMG**	**Heterozygous healthy family members**
1	*TNNT2*	chr1: 201334772 rs144900708	G → A c.260C>T p.Pro87Leu	missense	likely pathogenic (PM1, PP2, PM2, PP3)	I-2, II-2, II-4, II-6
2	*TTN*	chr2: 179635354 rs752069599	T → C c.8165A>G p.Gln2722Arg	missense	VUS (PM2, PP3)	I-1, II-1, II-5, II-6
3	*TTN*	chr2: 179610733 rs148147002	C → T c.16394G>A p.Arg5465Gln	missense	VUS (PM2)	I-2, II-1, II-2, II-4, II-5

ACMG, American College of Medical Genetics and Genomics. VUS, a variant of uncertain significance.

Supplementary Table S4: Evaluating and prioritizing potential heterozygous candidate variants in patient II-3.

Number of variants after filtration	Criteria for negation – heterozygous variants
**14109 → 8624**	Zygosity → Heterozygous
**8624→ 399**	Presence in the general databases – 1KG, EVS, ExAC and gnomAD at frequencies > 0.1%
**399 → 135**	Presence in our internal laboratory Exome database of the Bedouin population at frequencies > 0.5%
**135 → 37**	Reported in the public databases – gnomAD and GeniePool (assuming de-novo variation)
**37 → 31**	Benign aggregated prediction by Franklin browser
**31 → 3**	Genes associated with different clinical presentations by OMIM and GeneCards
**3 → 0**	Low expression in the heart by Genotype-Tissue Expression (GTEx)

Supplementary Table S5: Evaluating and prioritizing potential homozygous candidate variants in patient II-3.

Number of variants after filtration	Criteria for negation – homozygous variants
**14109 → 5485**	Zygosity → Homozygous
**5485 → 81**	Presence in the general databases – 1KG, EVS, ExAC and gnomAD at frequencies > 0.1%
**81 → 12**	Presence in our internal laboratory Exome database of the Bedouin population at frequencies > 0.5%
**12 → 10**	Not expressed in the heart by Genotype-Tissue Expression (GTEx)
**10 → 7**	Low damage prediction scores (CADD < 10, Omicia Variant Score < 0.2, SIFT Score > 0.1)
**7 → 2**	Segregation analysis of healthy family members presenting variation in homozygosity
**2 → 1**	Non-cardiac phenotypes in literature

Supplementary Table S6: Full list of the 11 homozygous variants that were negated by cardiac expression, damage prediction scores, segregation analysis, and cardiac phenotypes in literature, as detailed in Supplementary Table 2. Positions are according to GRCh37/hg19.

**Negated by non-cardiac expression by Genotype-Tissue Expression (GTEx)**										
**No**	**Gene**	**Position dbSNP**	**Change**	**Effect**	**SIFT**	**PolyPhen**	**CADD**	**Omicia**	**ClinVar**	**ACMG**
1	*OTOA*	chr16:21728262 rs138141474	T → C c.1523T>C p.Val508Ala	missense	0.921	benign	0.43	0.156	benign	benign (BA1, BS1, BS2, BP6)
2	*CHST5*	chr16:75563571	G → C c.712C>G p.Pro238Ala	missense	0.743	benign	0.006	0.094	NR	VUS (PM2, BP4)
**Negated by low damage prediction scores (CADD < 10, PolyPhen (B), Omicia Variant Score < 0.2, SIFT Score > 0.1)**										
**No**	**Gene**	**Position dbSNP**	**Change**	**Effect**	**SIFT**	**PolyPhen**	**CADD**	**Omicia**	**ClinVar**	**ACMG**
1	*AOX1*	chr2:201477442	G → C c.1374G>C p.Glu458Asp	missense	0.856	benign	0.074	0.075	NR	VUS (PM2, BP4)
2	*FASN*	chr17:80039669 rs770333916	T → C c.6214A>G p.Thr2072Ala	missense	0.481	benign	0.005	0.057	NR	VUS (PM2, BP4)
3	*C22orf29*	chr22:19839274 rs146494789	A → G c.511T>C p.Phe171Leu	missense	0	benign	10.09	0.148	NR	VUS (PM2, BP4)
**Negated by segregation analysis of healthy family members presenting variation in homozygosity**										

No	Gene	Position dbSNP	Change	Effect	Omicia	Family member	ClinVar	ACMG
1	*KIAA0319*	chr6:24564456 rs769768664	T → C c.2405A>G p.Asp802Gly	missense	0.697	II-2	NR	VUS (PM2, BP4)
2	*C10orf126*	chr10:29169271 rs751579044	G → A c.415G>A p.Val139Met	missense	0.401	II-1, II-2	NR	VUS (PM2)
3	*TMC8*	chr17:76129571 rs759778569	C → T c.616C>T p.Leu206Phe	missense	0.876	II-2	NR	VUS (PM2)
4	*PRODH*	chr22:18912653 rs199907923	C → T c.578G>A p.Gly193Asp	missense	0.798	I-1	NR	VUS (PM2, BP4)
5	*FAM211B*	chr22:24982267	C → T c.535G>A p.Gly179Ser	missense	0.623	II-2, II-6	NR	VUS (PM2, BP4)

Negated by non-cardiac phenotypes in literature

No	Gene	Position dbSNP	Change	Effect	Omicia	ClinVar	ACMG
1	*WDR60*	chr7:158711458 rs555394154	C → T c.1819C>T p.Arg607Cys	missense	0.501	NR	VUS (PM2)

ACMG, American College of Medical Genetics and Genomics. NR, not reported. VUS, a variant of uncertain significance.

Videos Video of control and patient's fibroblasts through wound healing assay. Fibroblast movement was monitored using a time-lapse every 20 min for 67 hrs. in live-cell FluoView 1000 fluorescence microscope (OLYMPUS), with a 20× objective. The video was made by merging all images taken in 20 min time-lapse (200 photos in total).
